# Factors associated with type of footwear worn inside the house: a cross-sectional study

**DOI:** 10.1186/s13047-019-0356-8

**Published:** 2019-08-23

**Authors:** Alex L. Barwick, Jaap J. van Netten, Sheree E. Hurn, Lloyd F. Reed, Peter A. Lazzarini

**Affiliations:** 1 School of Health & Human Sciences, Southern Cross University, Southern Cross Drive, Bilinga, Queensland 4225 Australia; 2 School of Clinical Sciences, Queensland University of Technology, Brisbane, Queensland Australia; 3 Institute of Health and Biomedical Innovation, Queensland University of Technology, Brisbane, Queensland Australia; 4 Department of Rehabilitation, Amsterdam UMC, University of Amsterdam, Amsterdam Movement Sciences, Amsterdam, the Netherlands; 5 School of Public Health & Social Work, Queensland University of Technology, Brisbane, Queensland Australia; 6 Allied Health Research Collaborative, Metro North Hospital & Health Service, Brisbane, Queensland Australia

**Keywords:** Footwear, Inpatient, Age, Neuropathy, Diabetes, Sociodemographic, Slippers, Barefoot

## Abstract

**Background:**

In specific populations, including those at risk of falls or foot ulcers, indoor footwear is an important aspect of preventative care. This study aims to describe the indoor footwear worn most over the previous year in a sample representative of the Australian inpatient population, and to explore the sociodemographic, medical, foot condition and foot treatment history factors associated with the indoor footwear worn.

**Methods:**

This was a secondary analysis of data collected from inpatients admitted to five hospitals across Queensland, Australia. Sociodemographic information, medical history, foot conditions and foot treatment history were collected as explanatory variables. Outcomes included the self-reported type of indoor footwear (from 16 standard footwear types) worn most in the year prior to hospitalisation, and the category in which the self-reported footwear type was defined according to its features: ‘protective’, ‘non-protective’ and ‘no footwear’. Multivariate analyses determined explanatory variables independently associated with each type and category.

**Results:**

Protective footwear was worn by 11% of participants (including 4% walking shoes, 4% running shoes, 2% oxford shoes), and was independently associated with education above year 10 level (OR 1.78, *p* = 0.028) and having had foot treatment by a specialist physician (5.06, *p* = 0.003). Most participants (55%) wore non-protective footwear (including 21% slippers, 15% thongs/flip flops, 7% backless slippers), which was associated with older age (1.03, *p* < 0.001). No footwear was worn by 34% of participants (30% barefoot, 3% socks only). Those of older age (0.97, p < 0.001) and those in the most disadvantaged socioeconomic group (0.55, *p* = 0.019) were less likely to wear no footwear (socks or barefoot).

**Conclusions:**

Only one in nine people in a large representative inpatient population wore a protective indoor footwear most of the time in the previous year. Whilst having education levels above year 10 and having received previous foot treatment by a specialist physician were associated with wearing protective footwear indoors, the presence of a range of other medical and foot conditions were not. These findings provide information to enable clinicians, researchers and policymakers to develop interventions aimed at improving indoor footwear habits that may help prevent significant health burdens such as falls and foot ulcers.

**Electronic supplementary material:**

The online version of this article (10.1186/s13047-019-0356-8) contains supplementary material, which is available to authorized users.

## Background

Footwear protects the feet from the environment, provides a supportive surface between the feet and the ground [1] and has psychological and sociocultural significance [2, 3]. Some specific populations also have special footwear needs because of health conditions that increase their risk of experiencing falls [4] or developing foot ulcers, such as those with diabetes [5]. For example, older people at risk of falls are advised to wear footwear with a low heel, large sole contact area, secure fixation and a firm slip resistant sole [4]. However, regularly wearing footwear in line with that advice is complicated by contextual and personal factors [2]. Aesthetic preferences [6], financial priorities [3, 7], comfort [6, 7], presence of foot problems [3, 8] are all influencing factors in footwear decision-making [3, 6–8]. Some of these may be differently prioritised in footwear worn inside the home, compared to those worn outside the home, for example, people are willing to spend less on their indoor footwear [7].

As people at high risk of diabetic foot ulceration do most of their weight-bearing activity indoors [9] and many falls in the elderly occur indoors [10], choice of indoor footwear, in particular, has the potential to have significant impacts on these health outcomes. Prior studies examining the indoor footwear worn by specific populations at risk of falls or foot ulcers suggests that the everyday indoor footwear they wear is far from optimal. Older people and those at risk of falls due to Parkinson’s disease or stroke have been reported to commonly wear slippers or go without footwear altogether in the home [7, 8, 11]. This is similar for people with diabetes and a history of foot ulceration [12], and importantly, those who have been prescribed footwear to prevent foot ulceration often fail to wear these inside the home [9].

To our knowledge, no population-based study has examined the different indoor footwear worn and the factors associated with this in a large representative sample. This paper aims to investigate the indoor footwear types worn most often in the year prior to hospitalisation in a large representative inpatient population and the proportion of this footwear that falls within three overarching footwear categories: ‘protective footwear’, ‘non-protective footwear’ and ‘no footwear’ based on recognised guidelines [5, 13]. Further, the paper will explore the sociodemographic, medical condition, foot condition and foot treatment factors associated with wearing different indoor footwear types and categories. Such population-based information identifying potential moderating factors in such strategies may assist clinicians, researchers and public health policymakers to target strategies that reduce footwear-related health risks and their associated burden.

## Methods

This study was a secondary analysis of data from the *Foot Disease in Inpatients Study*, which has been described in detail elsewhere [14, 15] and in summary below. Participants were recruited from five public hospitals in Queensland (Australia). All adult inpatients admitted for any medical reason and at least one night hospital stay were eligible for the study, except those in a maternity or psychiatric ward or those with a cognitive deficit. Overall, 733 of the 883 eligible inpatients present in those hospitals agreed to participate and provided voluntary informed consent. This inpatient sample has been reported to be highly representative of the sociodemographic characteristics and the co-morbidities of the general Australian adult inpatient population: mean (SD) age 62 [16], 56% (*n* = 408) male, 61% (*n* = 435) residing in a major city, 22% (*n* = 161) born overseas, 5% (*n* = 34) indigenous [14, 15]. The Prince Charles Hospital Human Research Ethics Committee (HREC) (Ethics No. HREC/13/QPCH/5) and the Queensland University of Technology HREC (Ethics No. 1300000367) approved this study along with approval from each hospital.

Self-reported explanatory variables were collected for each participant by trained data collectors who were registered podiatrists and recorded on a validated data collection form [14, 15, 17]. This previously validated form, the Queensland High Risk Foot Form, was designed to capture foot risk factors and complications in diverse populations [17]. The variables included: sociodemographic factors (age, sex, indigenous status, country of birth, socioeconomic status (using the participant’s postcode of usual residence and the Australian Index of Relative Social Disadvantage to determine [18]), geographical remoteness (using the participant’s postcode of usual residence and the Accessibility/Remoteness Index of Australia status to determine [19])), medical history (diabetes, hypertension, dyslipidaemia, myocardial infarct, stroke, chronic kidney disease, cancer, arthritis, depression, smoking, mobility impairment, vision impairment), and foot treatment in the year prior to hospitalisation (by podiatrist, general medical practitioner, specialist (non-general medical practitioner) physician, surgeon, nurse, orthotist or other) [17].

Trained data collectors clinically examined the feet of participants to diagnose foot conditions. These included: lower extremity amputation (performed during current or previous admission), foot ulcer(s) (active or previous), peripheral neuropathy, peripheral artery disease (PAD) and foot deformities. In brief, peripheral neuropathy was diagnosed as the failure to sense a 10-g monofilament on at least two plantar forefoot sites on one foot [16, 20]. PAD was diagnosed when toe systolic pressure was < 70 mmHg. Severity of PAD was classified as mild (51-70 mmHg), moderate (31-50 mmHg) or critical (< 30 mmHg) [21, 22]. Foot deformity was diagnosed when three or more of the following characteristics were present on one foot: small muscle wastage, bony prominence, prominent metatarsal heads, hammer or claw toes, limited joint mobility or Charcot deformity [16].

The outcome of interest for this study was footwear worn most of the time in the house in the previous year. Each participant was presented with a validated footwear picture chart [23] modified to add a barefoot option and a socks only option. The participant was asked “from this chart displaying 16 different types of footwear, what is the one type of shoe you have worn most inside the house over the past 12 months?” The previous 12 months was chosen as a time period as this is most relevant to the participant’s current health and sociodemographic situation whilst also being within the participant’s recall. Seven participants had missing data for this outcome variable and were removed, thus a population of 726 participants were used for this study.

The type of indoor footwear selected by participants was then categorised into three broad footwear categories: ‘protective footwear’, ‘non-protective footwear’ and ‘no footwear’. These categories were based on recommended protective features deducible from the footwear type selected. Those protective footwear features were recommended by the Expert Group Criteria for the Recognition of Healthy Footwear [13] and Diabetic Foot Australia footwear guidelines [5] and included: a stable heel, a heel pitch no more than 2.5 cm, an enclosed upper, minimal torsional flexibility and a fixation system. ‘Protective footwear’ included walking shoes, running shoes, oxford shoes, boots, and bespoke footwear. ‘Non-protective footwear’ was defined as footwear types that lacked the above protective features and included moccasins, Ugg boots, high heels, thongs/flip flops, slippers, backless slippers, court shoes, mules, and sandals. The ‘no footwear’ category included barefoot or socks only.

### Statistical analysis

All data were analysed using SPSS 23.0 for Windows (SPSS Inc., Chicago, IL, USA) or GraphPad Prism (GraphPad Software Inc., San Diego, CA, USA). Descriptive statistics were used to display all variables. Prevalence with 95% Confidence Intervals (95% CI) was evaluated for the three footwear groups as well as each of the 16 indoor footwear types. Associations between explanatory variables and the footwear categories and types were analysed using univariate logistic regression.

All explanatory variables achieving a statistical significance of *p* < 0.2 with outcome variables were included in backwards stepwise multivariate logistic regression analysis until only variables reaching statistical significance remained (*p* < 0.05) (Unadjusted Model) [14, 24, 25]. The unadjusted model was then adjusted for age, sex, socio-economic status and geographical remoteness by entering these variables into the model with the variables remaining in the unadjusted model (Adjusted Model) [14, 24, 25]. Collinearity, goodness of fit, significance, parsimony and variance were assessed at each step and found to be acceptable. Cases with missing data were excluded, as the proportion of missing data cases was minimal (< 5% in all cases) [14, 24, 25]. Please note only footwear categories and individual footwear types with a prevalence of > 1% were tested in the multivariate models.

## Results

Table 1 displays the prevalence of each of the three footwear categories and each footwear type within that category. Protective footwear was worn by 11% of participants most of the time in the previous year with the most common types being walking shoes at 4% and running shoes at 4% of the total sample. Non-protective footwear was worn by 55% of participants with the most common types being slippers at 21%, thongs/flip flops at 15% and backless slippers at 7%. Further, moccasins were worn by 5% and sandals were worn by 4% of the sample. Finally, wearing no footwear indoors most of the time in the previous year was worn by 34% including 30% barefoot and 3% socks only.

**Table 1:** Prevalence of footwear categories and types worn inside the house most of the time in the previous year (*n* = 726)

Category	Type	n	% (95% CI)
Protective	Walking shoes	31	4% (3.0–6.0)
	Running shoes	28	4% (2.7–5.5)
	Oxford shoes	11	2% (0.8–2.7)
	Bespoke footwear	6	< 1% (0.3–1.8)
	Boots	5	< 1% (0.3–1.7)
Total		81	11% (9.1–13.7)
Non-protective	Slippers	155	21% (18.5–24.5)
	Thongs/flip flops	105	15% (12.1–17.2)
	Backless slippers	54	7% (5.7–9.6)
	Moccasins	34	5% (3.4–6.4)
	Sandals	30	4% (2.9–5.9)
	Ugg Boots	19	3% (1.7–4.1)
	Court shoes	4	< 1% (0.2–1.5)
	Mules	0	0
	High heels	0	0
Total		401	55% (51.6–58.8)
No Footwear			
	Barefoot	219	30% (26.9–33.6)
	Socks only	25	3% (2.3–5.1)
Total		244	34% (30.3–37.1)

Additional file 1 displays the prevalence and univariate analyses for each footwear category and Additional file 2, Additional file 3, Additional file 4 and Additional file 5 display the univariate analyses for each footwear type. Table 2 shows the unadjusted and adjusted multivariate associations with each footwear category and Table 3 displays this data for each footwear type.

**Table 2:** Independent factors associated with three categories of footwear type worn most inside the house in the previous year (Odds Ratios [95% CI])

Category	Risk Factor	Unadjusted	*p* Value	Adjusted^a^	*p* Value
Protective	>Year 10 Education	1.70 [1.06–2.72]	0.027*	1.78 [1.06–2.99]	0.028*
	Past Specialist Treatment	3.28 [1.23–8.76]	0.018*	5.06 [1.75–14.63]	0.003*
Non-protective	Age (year)	1.03 [1.02–1.04]	< 0.001*	1.03 [1.02–1.04]	< 0.001*
No footwear	Age (year)	0.97 [0.96–0.98]	< 0.001*	0.97 [0.96–0.98]	< 0.001*
	Socioeconomic Status		0.021*		0.028*
	Least disadvantaged	1.00		1.00	
	Second least disadvantaged	1.09 [0.62–1.92]	0.775	1.05 [0.56–1.94]	0.888
	Middle	0.94 [0.56–1.57]	0.797	0.89 [0.52–1.53]	0.666
	Second most disadvantaged	0.57 [0.31–1.04]	0.066	0.52 [0.27–1.01]	0.052
	Most disadvantaged	0.57 [0.34–0.93]	0.024*	0.55 [0.34–0.91]	0.019*

^a^Adjusted for age, sex, socio-economic status and geographical remoteness *p < 0.05; Missing: Excluded missing cases

**Table 3:** Independent factors associated with footwear type worn most inside the house in previous year (Odds Ratios [95% CI])

	Risk Factor	Unadjusted	*p* Value	Adjusted^a^	*p* Value
PROTECTIVE					
Walking Shoes	Nil	–		–	
Running Shoes	No diabetes	3.63 [1.04–12.72]	0.044*	4.11 [1.10–15.38]	0.036*
	Stroke	3.02 [1.27–7.18]	0.012*	3.70 [1.43–9.57]	0.007*
	Peripheral Neuropathy	2.57 [1.12–5.90]	0.026*	3.40 [1.28–8.99]	0.014*
Oxford Shoes	Born overseas	4.32 [1.30–14.34]	0.017*	3.85 [1.13–13.13]	0.031*
NON-PROTECTIVE					
Slippers	Age (year)	1.06 [1.05–1.08]	< 0.001*	1.07 [1.05–1.08]	< 0.001*
	<Year 10 Education Level	1.80 [1.17–2.76]	0.007*	1.83 [1.19–2.82]	0.006*
	Socioeconomic Status		< 0.001*		< 0.001*
	Most disadvantaged	1.00		1.00	
	Second most disadvantaged	2.20 [1.09–4.42]	0.028*	2.33 [1.14–4.73]	0.020*
	Middle	0.94 [0.42–2.14]	0.888	1.08 [0.45–2.60]	0.869
	Second least disadvantaged	2.54 [1.31–4.91]	0.006*	2.76 [1.33–5.74]	0.006*
	Least disadvantaged	0.54 [0.22–1.34]	0.184	0.58 [0.23–1.49]	0.256
Thongs/Flip Flops	Smoker	2.81 [1.65–4.78]	< 0.001*	2.11 [1.20–3.73]	0.010*
	No mobility impairment	5.35 [2.69–10.66]	< 0.001*	4.21 [2.06–8.60]	< 0.001*
	Geographical Remoteness		0.004*		0.019*
	Major city	1.00		1.00	
	Inner regional area	1.08 [0.61–1.92]	0.794	1.06 [0.55–2.04]	0.868
	Outer regional area	1.43 [0.70–2.94]	0.328	1.46 [0.68–3.13]	0.329
	Remote area	4.46 [1.89–10.50]	0.001*	4.38 [1.73–11.05]	0.002*
	Very remote area	2.83 [1.09–7.39]	0.033*	2.68 [0.90–7.93]	0.075
Backless Slippers	Female	2.21 [1.23–3.95]	0.008*	2.19 [1.2–3.96]	0.009*
	Born overseas	2.93 [1.62–5.30]	< 0.001*	3.09 [1.69–5.66]	< 0.001*
	Hypertension	2.19 [1.21–3.97]	0.009*	2.07 [1.09–3.93]	0.026*
Moccasins	Age (years)	1.04 [1.01–1.06]	0.002*	1.03 [1.01–1.06]	0.008*
	Past Orthotist Treatment	19.65 [1.26–306.3]	0.034*	26.94 [1.40–519.6]	0.029*
	No foot deformity	3.30 [1.12–9.76]	0.031*	4.09 [1.19–14.11]	0.026*
Sandals	Nil	–		–	
Ugg Boots	Female	2.79 [1.05–7.43]	0.040*	2.82 [1.05–7.60]	0.040*
NO FOOTWEAR					
Barefoot	Age (year)	0.97 [0.96–0.98]	< 0.001*	0.97 [0.96–0.98]	< 0.001*
	Stroke history	0.38 [0.19–0.76]	0.007*	0.38 [0.19–0.77]	0.007*
	Socioeconomic Status		0.018*		0.043*
	Least disadvantaged	1.00		1.00	
	Second least disadvantaged	1.13 [0.63–2.04]	0.680	1.04 [0.55–1.99]	0.896
	Middle	1.08 [0.63–1.84]	0.784	0.98 [0.56–1.73]	0.955
	Second most disadvantaged	0.64 [0.34–1.21]	0.173	0.59 [0.30–1.17]	0.131
	Most disadvantaged	0.57 [0.34–0.95]	0.030*	0.55 [0.33–0.93]	0.025*
Socks only	Depression	0.11 [0.02–0.85]	0.034*	0.12 [0.02–0.87]	0.036*

^a^Adjusted for age, sex, socio-economic status and geographical remoteness *p < 0.05; Missing: Excluded missing cases

### Protective footwear

In the adjusted multivariate analyses, wearing protective footwear most of the time indoors was independently associated (Odds Ratio; 95% CI) with an education level above year 10 (1.78; 1.06–2.99, *p* = 0.028) and having had foot treatment by a specialist physician in the previous year (5.06; 1.75–14.63, *p* = 0.003). Those wearing walking shoes were not found to be independently associated with any variable. Wearing running shoes was independently associated with not having diabetes (4.11; 1.10–15.38, *p* = 0.036), having a history of stroke (3.7; 1.43–9.57, *p* = 0.007) and having peripheral neuropathy (3.4; 1.28–8.99, *p* = 0.014). Wearing oxford shoes was independently associated with being born overseas (3.85; 1.13–13.13, *p* = 0.031).

### Non-protective footwear

Wearing non-protective footwear most of the time in the house was independently associated with older age (1.03 per year; 1.02–1.04, *p* < 0.001) in the adjusted multivariate analyses. Wearing slippers was independently associated with older age (1.07 per year; 1.05–1.08, *p* < 0.001), an education level below year 10 (1.83; 1.19–2.82, *p* = 0.006) and the second most (2.33; 1.14–4.73, *p* = 0.02) and second least disadvantaged (2.76; 1.33–5.74, *p* = 0.006) socioeconomic groups. Wearing thongs/flip flops was independently associated with being a smoker (2.11; 1.2–3.73, *p* = 0.010), not having a mobility impairment (4.21; 2.06–8.60, *p* < 0.001), and living in a remote area (4.38; 1.73–11.05, *p* = 0.002). Wearing backless slippers was independently associated with being female (2.19; 1.2–3.96, *p* = 0.009), being born overseas (3.09; 1.69–5.66*, p* < 0.001) and having hypertension (2.07; 1.09–3.93, *p* = 0.026). Wearing moccasins was independently associated with older age (1.03 per year; 1.01–1.06, *p* = 0.008) and previous foot treatment by an orthotist (26.94, 1.4–519.6, *p* = 0.029) and not having a foot deformity (4.09; 1.19–14.11, *p* = 0.026). Wearing sandals indoors was not independently associated with any of the variables and wearing Ugg boots was independently associated with being female (2.82; 1.05–7.6, *p* = 0.04).

### No footwear

Older people (0.97 per year of age; 0.96–0.98, *p* < 0.001) and the most disadvantaged socioeconomic group were less likely to wear no footwear (0.55; 0.34–0.91, *p* = 0.019) in the adjusted multivariate analysis. Older people (0.97 per year; 0.96–0.98, *p* < 0.001), those in the most disadvantaged socioeconomic group (0.55; 0.33–0.93, *p* = 0.025) and those with a history of stroke (0.38; 0.19–0.77, *p* = 0.007) were less likely to go barefoot.. Those with depression were less likely to wear socks only (0.12; 0.02–0.87, *p* = 0.036).

## Discussion

This cross-sectional study is, to the best of our knowledge, the first to examine indoor footwear worn most of the time in the past year by a large sample representative of the Australian inpatient population. Inside the house, the categories of footwear that were worn most of the time for the past 12 months were either non-protective footwear such as slippers and thongs/flip flops, or no footwear at all, collectively representing almost 90% of the sample. Although indoor footwear is considered important in a number of health conditions, only a small proportion of people wore indoor footwear considered to be protective. We found some interesting independent associations between different indoor footwear categories (protective, non-protective and no footwear) and various sociodemographic, medical history, and foot treatment history variables, as well as further associations between these variables and individual footwear types in our sample.

Only one in nine people wore a protective indoor footwear type. Wearing such protective footwear was not independently associated with medical or foot conditions that normally require protective footwear, such as peripheral neuropathy or history of amputation. This demonstrates the disconnection between footwear recommendations and actual footwear use in these populations. This is often acknowledged clinically and has been demonstrated previously in people with a history of stroke, Parkinson’s disease [8] and diabetic foot ulceration [12]. Our finding that one in nine people wear protective footwear inside the house indicates that this disconnection may be much more distinct in footwear worn inside the house than that worn outside the house with close to one in two people wearing protective footwear outside the house reported in our previous paper [26].

This is consistent with prior research in people at high risk of ulceration who have been found to be more likely to adhere to their prescribed bespoke shoes outside the house than inside the house [9]. This highlights the importance of specifically inquiring about footwear habits inside the house and implementing both indoor and outdoor footwear-related preventative measures in clinical encounters with at-risk patients. This is particularly pertinent when considering some of these populations have been shown to do more weight-bearing activity indoors than they do outdoors [9].

Previous foot care by a specialist medical physician in the previous year was also associated with protective footwear. Yet, foot care by any other health professional (podiatrist, general practitioner, surgeon, nurse, orthotist, other) was not associated with wearing protective footwear. This was against our expectations, as we would expect footwear change interventions to be implemented successfully by most of these health professionals. This lack of association along with the high proportion of those with risk factors not wearing protective footwear inside the home, demonstrates a lack of implementation of effective footwear change interventions, particularly for footwear worn inside the house. Recently, motivational interviewing has been demonstrated to increase the adherence to therapeutic footwear in those at high risk of ulceration in the short-term [27]. Though more research on effective strategies to reduce footwear related risk are required, it is recommended that consideration to the practicalities, purpose and social norms are considered [28]. Further to this, wearing protective footwear was independently associated with an education level above year 10. This relationship may be mediated by the link between lower educational attainment and poorer health literacy [29] so this should be considered in the delivery of indoor footwear. The translatability of current footwear guidelines to clinical practice and acceptability of such footwear to patients is also a topic for further research.

The specific protective footwear types most worn were walking shoes and running shoes. Wearing walking shoes was not independently associated with any factors. However, wearing running shoes was independently associated with peripheral neuropathy, in line with recommendations for this group, but conversely running shoes were also much more likely to be worn by those without diabetes which contradicts the recommendations for this group [5]. Furthermore, wearing running shoes was independently associated with history of stroke, whilst not going barefoot was associated with having a history of stroke which is consistent with previous research [8]. Bowen et al. [8] found that following a stroke or Parkinson’s diagnosis people change their indoor footwear towards being more supportive suggesting this is for reasons of foot problems and mobility changes. However, the overall proportion of those with a history of stroke in our sample who reported wearing protective footwear most of the time inside the house in the previous year was low at only 18%. The final association found with a protective footwear type was oxford shoes being associated with being born overseas. This highlights potential cultural factors influencing footwear preferences.

Non-protective footwear was only associated with older age with odds increasing by 3% per year of age. A form of slippers (either backless slippers or standard slippers) were the most common non-protective footwear type worn in our study, but this was still a lower proportion, at 28%, than that of previous studies. Munro and Steele [7] found up to 38% of people over 65 living in the community wore slippers indoors, and Davis et al. [6] found 48% of women aged between 60 and 80 years also preferred wearing slippers indoors. This is potentially explained by our sample being more diverse in age (18–99), as slippers too were associated with age with odds increasing by 7% with each year of age and perhaps a warmer climate of Queensland Australia compared with New South Wales and Victoria where these previous studies were performed. Slippers have been found to be unsupportive, quick to lose their structural integrity and can have a lack of grip and fixation [7]. They are therefore not recommended for populations at risk of falls or foot ulcers, although more research is needed into the types of footwear that contributes to or prevents falls [30].

Wearing slippers was also independently associated with other sociodemographic factors including an education level below year 10 and socio-economic status, whereas backless slippers were associated with being female. Interestingly, female gender does not appear to be as much of a predictor of indoor footwear types as it is of outdoor footwear, as identified in our previous paper [26]. Only backless slippers and Ugg boots were independently associated with female gender in this study compared to seven types of outdoor footwear in our previous paper. This may be reflective of the more homogenous footwear types worn in the home compared to outdoors.

Thongs/flip flops were the second most common non-protective footwear type, being worn by 15% of the sample. This was similar to the 13% finding by Munro and Steele [7] in older people. We also found that those with a mobility impairment were less likely to wear thongs/flip flops in keeping with a finding by Bowen et al. [8] that people with stroke and Parkinson’s almost never wore thongs/flip flops indoors. The strongest association with wearing thongs/flip flops was living in a remote area, while smoking was also independently associated. Moccasins were also associated strongly with having seen an orthotist, age and not having a deformity. However, since < 5% of the sample wore moccasins these associations should be interpreted with caution.

The predominance of slippers and thongs/flip flops fits with what we know about considerations when purchasing indoor footwear in some specific populations such as older people and women with rheumatoid arthritis, where comfort and the convenience of not bending down to don and doff are important [3, 7]. The large proportion of people preferring to go without footwear in the home is also perhaps not surprising, especially given the warm climate of Queensland, Australia from which this sample was drawn. Further, there are sometimes cultural and religious reasons for not wearing footwear or wearing slip-on footwear in some indoor areas [3, 31]. These contextual factors should be considered when making clinical recommendations.

The factor that associated most often with indoor footwear type and category worn most in the previous year was age. In addition to the previously mentioned associations between increasing age and non-protective footwear, slippers and moccasins, older people were less likely to go barefoot and this relationship was maintained when adding socks in with barefoot in our ‘no footwear’ group. This also has implications for falls prevention as going barefoot is associated with falls in older people [32]. The variety of relationships between footwear types and age may reflect generational preferences, or perhaps as people age and develop chronic health conditions they may value the warmth and comfort that wearing footwear such as slippers and moccasins in the home can provide compared to being barefoot. The finding that older people are more likely to wear non-protective footwear like slippers and moccasins demonstrates the need for behaviour change strategies around footwear in this population. However, the finding that older people are less likely to go without footwear is a positive starting point that may enable behaviour change interventions to more easily facilitate a move towards more protective indoor footwear.

The results of this analysis should be interpreted in the context of some strengths and limitations. The strengths of this study include that the sample was large and reported to be highly representative of an Australian inpatient population. It provides for the first time insights into the footwear habits of a large population-based sample and the sociodemographic, health and foot-related associations. The data collection instruments have demonstrated validity and reliability [17]. However, the study is at risk of type 1 error with the volume of analyses performed, and this may account for some of the associations we identified that have seemingly no potential causal pathway or other explanation for the association; for example, not wearing socks only was associated with having depression, which does not seem to have a plausible explanation. Further, the outcome was determined through the footwear type worn most often inside the house in the previous year, which is subject to recall bias and also does not represent all indoor footwear use. Our categorisation of footwear was based on recognised guidelines [5, 13], however, there are some recommendations that were not able to be determined just from the footwear type selected. For example, the fit of the footwear on the wearer could not be assessed for appropriateness. Further, our categories of protective footwear, non-protective footwear and no footwear based on recommendations and guidelines assumes that the individual shoe was characteristic of the footwear type.

## Conclusions

We found that most people in a large representative population-based inpatient sample in Queensland (Australia) wear non-protective footwear such as thongs/flip flops or slippers or go barefoot most of the time indoors. We did not find those with foot problems or those who had foot care by most providers to be more likely to employ safe footwear habits inside the home. The findings of this study demonstrate the need to focus attention on indoor footwear use as particularly problematic in these at-risk populations Sociodemographic factors such as education level and age are also associated with footwear and should be considered in any intervention that aims to change footwear habits. Further longitudinal research should examine the suggested reasons for these associations found in this cross-sectional analysis, including any causal relationships and how they may modify treatment and education strategies. The observations of this study should help inform clinicians, researchers and policymakers to develop interventions aimed at improving indoor footwear habits that may help prevent significant health care burdens such as falls and foot ulcers in future.

## Additional files

Additional file 1: **Table S1.** Characteristics and univariate analysis for those participants mostly wearing three categories indoor footwear types of no footwear, unprotective footwear and protective footwear. (DOCX 78 kb)

Additional file 2: **Table S2.** Characteristics and univariate analysis for those participants mostly wearing the indoor footwear types of barefoot, slippers, thongs/flip flops. (DOCX 78 kb)

Additional file 3: **Table S3.** Characteristics and univariate analysis for those participants mostly wearing the indoor footwear types of backless slippers, moccasins, walking shoes. (DOCX 78 kb)

Additional file 4: **Table S4.** Characteristics and univariate analysis for those participants mostly wearing the indoor footwear types of sandals, running shoes, socks only. (DOCX 77 kb)

Additional file 5: **Table S5.** Characteristics and univariate analysis for those participants mostly wearing the indoor footwear types of ugg boots, oxford shoes. (DOCX 25 kb)

## Data Availability

All data is publicly available in our institutions research data repository. The details are: Citation: Lazzarini, Peter (2019): Dataset of the Foot Disease in Inpatients Study. Queensland University of Technology. (Dataset) 10.25912/5c53a22ae2810; DOI: 10.25912/5c53a22ae2810; URL: https://researchdatafinder.qut.edu.au/display/n1802.

## Abbreviations

CI: Confidence interval
HREC: Human Research Ethics Committee
PAD: peripheral arterial disease

## References

[1] McPoil TG (1988). Footwear. Phys Ther.

[2] Nicholls E, Robinson V, Farndon L, Vernon W (2018). A good fit?‘bringing the sociology of footwear to the clinical encounter in podiatry services: a narrative review. JFAR..

[3] Tehan PE, Morpeth T, Williams AE, Dalbeth N, Rome K (2019). “Come and live with my feet and you’ll understand” – a qualitative study exploring the experiences of retail footwear in women with rheumatoid arthritis. JFAR..

[4] Menant JC, Steele JR, Menz HB, Munro BJ, Lord SR (2008). Optimizing footwear for older people at risk of falls. J Rehabil Res Dev.

[5] van Netten JJ, Lazzarini PA, Armstrong DG, Bus SA, Fitridge R, Harding K (2018). Diabetic foot Australia guideline on footwear for people with diabetes. JFAR.

[6] Davis A, Murphy A, Haines TP (2013). "Good for older ladies, not me": how elderly women choose their shoes. JAPMA.

[7] Munro BJ, Steele JR (1999). Household-shoe wearing and purchasing habits. A survey of people aged 65 years and older. JAPMA.

[8] Bowen C, Ashburn A, Cole M, Donovan-Hall M, Burnett M, Robison J (2016). A survey exploring self-reported indoor and outdoor footwear habits, foot problems and fall status in people with stroke and Parkinson's. JFAR.

[9] Waaijman R, Keukenkamp R, de Haart M, Polomski WP, Nollet F, Bus SA (2013). Adherence to wearing prescription custom-made footwear in patients with diabetes at high risk for plantar foot ulceration. Diabetes Care.

[10] Gelbard R, Inaba K, Okoye OT, Morrell M, Saadi Z, Lam L (2014). Falls in the elderly: a modern look at an old problem. Am J Surg.

[11] Vass C, Edwards C, Smith A, Sahota O, Drummond A (2015). What do patients wear on their feet? A service evaluation of footwear in elderly patients. Int J Ther Rehab.

[12] Reiber GE, Smith DG, Wallace CM, Vath CA, Sullivan K, Hayes S (2002). Footwear used by individuals with diabetes and a history of foot ulcer. J Rehabil Res Dev.

[13] Vernon W, Borthwick AM, Walker J, Hardy B, Dunning D, Denton C (2007). Expert group criteria for the recognition of healthy footwear. Br J Podiatr.

[14] Lazzarini PA, Hurn SE, Kuys SS, Kamp MC, Ng V, Thomas C (2016). Direct inpatient burden caused by foot-related conditions: a multisite point-prevalence study. BMJ Open.

[15] Lazzarini PA, Hurn SE, Kuys SS, Kamp MC, Ng V, Thomas C (2017). The silent overall burden of foot disease in a representative hospitalised population. Int Wound J.

[16] Baker, IDI. National Evidence-Based Guideline on Prevention, Identification and Management of Foot Complications in Diabetes (Part of the Guidelines on Management of Type 2 Diabetes). Edited by Baker IDI. Melbourne, Australia: Commonwealth of Australia; 2011.

[17] Lazzarini PA, Ng V, Kinnear EM, Kamp MC, Kuys SS, Hurst C (2014). The Queensland high risk foot form (QHRFF)–is it a reliable and valid clinical research tool for foot disease?. JFAR.

[18] Australian Bureau of Statistics (ABS). Information paper: an introduction to socio-economic indexes for areas (ABS Cat. No. 2039.0). Canberra: ABS, 2006. http://www.abs.gov.au/ausstats/abs@.nsf/mf/2039.0/. (accessed 28 Aug 2015).

[19] Australian Bureau of Statistics (ABS). Australian Standard Geographical Classification (ABS Cat No. 1216.0). Canberra: ABS, 2011. http://www.abs.gov.au/ausstats/abs@.nsf/mf/1216.0. (accessed 28 Aug 2015).

[20] Schaper NC (2004). Diabetic foot ulcer classification system for research purposes: a progress report on criteria for including patients in research studies. Diabetes Metab Res Rev.

[21] Jeffcoate WJ, Bus SA, Game FL, Hinchliffe RJ, Price PE, Schaper NC (2016). Reporting standards of studies and papers on the prevention and management of foot ulcers in diabetes: required details and markers of good quality. Lancet Diabetes Endocrinol.

[22] Mills JL, Conte MS, Armstrong DG, Pomposelli FB, Schanzer A, Sidawy AN (2014). The society for vascular surgery lower extremity threatened limb classification system: risk stratification based on wound, ischemia, and foot infection (WIfI). J Vasc Surg.

[23] Barton CJ, Bonanno D, Menz HB (2009). Development and evaluation of a tool for the assessment of footwear characteristics. JFAR.

[24] Hosmer Jr DW, Lemeshow S, Sturdivant RX. Applied logistic regression. 2nd edn. New York: Wiley; 2013.

[25] Tabachnick BG, Fidell LS (2013). Using multivariate statistics. 6 ed.

[26] Barwick A, van Netten JJ, Reed L, Lazzarini P. Independent factors associated with wearing different types of outdoor footwear in a representative inpatient population: a cross-sectional study JFAR 2018;11(1):19. 10.1186/s13047-018-0260-7 PMC5975543 29854004

[27] Keukenkamp R, Merkx MJ, Busch-Westbroek TE, Bus SAJJotAPMA. An explorative study on the efficacy and feasibility of the use of motivational interviewing to improve footwear adherence in persons with diabetes at high risk for foot ulceration. 2018;108(2):90–9. 10.7547/16-171 29111785

[28] Farndon L, Robinson V, Nicholls E, Vernon W (2016). If the shoe fits: development of an on-line tool to aid practitioner/patient discussions about 'healthy footwear'. JFAR.

[29] van der Heide I, Wang J, Droomers M, Spreeuwenberg P, Rademakers J, Uiters EJJohc. The relationship between health, education, and health literacy: results from the Dutch Adult Literacy and Life Skills Survey. 2013;18(sup1):172–84. 10.1080/10810730.2013.825668 PMC3814618 24093354

[30] Davis A, Haines T, Williams CJFS (2019). Do footwear styles cause falls or increase falls risk in healthy older adults? A systematic review. Footwear Science.

[31] Mazumdar S, Mazumdar S (2005). How organizations interface with religion: a typology. J Manag Spiritual Religion.

[32] Kelsey JL, Procter-Gray E, Nguyen U-SD, Li W, Kiel DP, Hannan MT (2010). Footwear and falls in the home among older individuals in the MOBILIZE Boston study. Footwear Science.

