# Supplementary material for: Factors associated with type of footwear worn inside the house: a cross-sectional study
Source: J Foot Ankle Res. 2019 Aug 23;12:45. doi: 10.1186/s13047-019-0356-8 (PMC6708142; doi:10.1186/s13047-019-0356-8)
Supplement: Supplementary file 1 — Table S1. Characteristics and univariate analysis for those participants mostly wearing three categories indoor footwear types of no footwear, unprotective footwear and protective footwear. (DOCX 78 kb) [file 13047_2019_356_MOESM1_ESM.docx]

**Additional file 1: Table S1:** Characteristics and univariate analysis for those participants mostly wearing **three categories** indoor footwear types of no footwear, unprotective footwear and protective footwear

| Variables | All | No Footwear | | | Non-protective Footwear | | | Protective Footwear | | |
| --- | --- | --- | --- | --- | --- | --- | --- | --- | --- | --- |
|  |  | No | Yes | *p* Value | No | Yes | *p* Value | No | Yes | *p* Value |
| **Participants** | 726 | 482 | 244 | 33.6% (30.3-37.1) | 325 | 401 | 55.2%  (51.6-58.5) | 645 | 81 | 11.2% (9.1-13.7) |
| **Socio-demographics** | 725 |  |  |  |  |  |  |  |  |  |
| Age (SD) or [IQR] years | 65(18) | 68 [56-80] | 58 [42-71] | <0.001** | 61 [43-73] | 68 [56-80] | <0.001** | 65 [50-76] | 67 [53-77] | 0.608 |
| Male sex^ | 403 (55.7%) | 265 (55.1%) | 138 (56.8%) | 0.664 | 189 (58.3%) | 214 (53.5%) | 0.193* | 352 (54.7%) | 51 (63.0%) | 0.161* |
| Indigenous | 34 (4.7%) | 22 (4.6%) | 12 (4.9%) | 0.836 | 16 (4.9%) | 18 (4.5%) | 0.789 | 30 (4.7%) | 4 (4.9%) | 0.911 |
| Born overseas^ | 161 (22.2%) | 110 (22.9%) | 51 (20.9%) | 0.538 | 72 (22.2%) | 89 (22.3%) | 0.961 | 140 (21.8%) | 21 (25.9%) | 0.397 |
| <Year 10 Education Level^ | 390 (53.9%) | 273 (56.8%) | 117 (48.1%) | 0.028** | 151 (46.4%) | 239 (59.8%) | <0.001** | 356 (55.4%) | 34 (42.0%) | 0.023** |
| Socioeconomic Status | 705 |  |  | 0.011** |  |  | 0.009** |  |  | 0.057* |
| Most disadvantaged | 101 (14.3%) | 60 (12.8%) | 41 (17.4%) |  | 46 (14.6%) | 55 (14.1%) |  | 96 (15.3%) | 5 (6.3%) |  |
| Second most disadvantaged | 157 (22.3%) | 98 (20.9%) | 59 (25.0%) |  | 74 (23.5%) | 83 (21.3%) |  | 142 (22.7%) | 15 (19.0%) |  |
| Middle | 97 (13.8%) | 71 (15.1%) | 26 (11.0%) |  | 40 (12.7%) | 57 (14.6%) |  | 83 (13.3%) | 14 (17.7%) |  |
| Second least disadvantaged | 238 (33.8%) | 174 (37.1%) | 64 (27.1%) |  | 90 (28.6%) | 148 (37.9%) |  | 212 (33.9%) | 26 (32.9%) |  |
| Least disadvantaged | 112 (15.9%) | 66 (14.1%) | 46 (19.5%) |  | 65 (20.6%) | 47 (12.1%) |  | 93 (14.9%) | 19 (24.1%) |  |
| Geographic Remoteness | 705 |  |  | 0.853 |  |  | 0.911 |  |  | 0.038** |
| Major city | 430 (61.0%) | 293 (62.5%) | 137 (58.1%) |  | 198 (62.9%) | 232 (59.5%) |  | 369 (58.9%) | 61 (77.2%) |  |
| Inner regional area | 152 (21.6%) | 97 (20.7%) | 55 (23.3%) |  | 66 (21.0%) | 86 (22.1%) |  | 141 (22.5%) | 11 (13.9%) |  |
| Outer regional area | 66 (9.4%) | 43 (9.2%) | 23 (9.7%) |  | 27 (8.6%) | 39 (10.0%) |  | 62 (9.9%) | 4 (5.1%) |  |
| Remote area | 30 (4.3%) | 19 (4.1%) | 11 (4.7%) |  | 13 (4.1%) | 17 (4.4%) |  | 28 (4.5%) | 2 (2.5%) |  |
| Very remote area | 27 (3.8%) | 17 (3.6%) | 10 (4.2%) |  | 11 (3.5%) | 16 (4.1%) |  | 26 (4.2%) | 1 (1.3%) |  |
| **Medical condition history** | 726 |  |  |  |  |  |  |  |  |  |
| Diabetes | 171 (23.6%) | 115 (23.9%) | 56 (23.0%) | 0.785 | 76 (23.4%) | 95 (23.7%) | 0.923 | 151 (23.4%) | 20 (24.7%) | 0.798 |
| Hypertension | 354 (48.8%) | 247 (51.2%) | 107 (43.9%) | 0.060* | 142 (43.7%) | 212 (52.9%) | 0.014** | 319 (49.5%) | 35 (43.2%) | 0.289 |
| Dyslipidaemia | 233 (32.1%) | 169 (35.1%) | 64 (26.2%) | 0.016** | 90 (27.7%) | 143 (35.7%) | 0.022** | 207 (32.1%) | 26 (32.1%) | 1.000 |
| Myocardial Infarct | 145 (20.5%) | 106 (22.0%) | 39 (16.0%) | 0.056* | 55 (16.9%) | 90 (22.4%) | 0.064* | 129 (20.0%) | 16 (19.8%) | 0.958 |
| Cerebrovascular Accident | 85 (11.7%) | 70 (14.5%) | 15 (6.1%) | 0.001** | 30 (9.2%) | 55 (13.7%) | 0.062* | 70 (10.9%) | 15 (18.5%) | 0.043** |
| Chronic Kidney Disease | 88 (12.1%) | 68 (14.1%) | 20 (8.2%) | 0.021** | 32 (9.8%) | 56 (14.0%) | 0.091* | 76 (11.8%) | 12 (14.8%) | 0.431 |
| Cancer | 171 (23.6%) | 116 (24.1%) | 55 (22.5%) | 0.647 | 72 (22.2%) | 99 (24.7%) | 0.424 | 154 (23.9%) | 17 (21.0%) | 0.564 |
| Arthritis | 270 (37.2%) | 194 (40.2%) | 76 (31.1%) | 0.017** | 101 (31.1%) | 169 (42.1%) | 0.002** | 245 (38.0%) | 25 (30.9%) | 0.211 |
| Depression | 189 (26.0%) | 130 (27.0%) | 59 (24.2%) | 0.418 | 84 (25.8%) | 105 (26.2%) | 0.918 | 164 (25.4%) | 25 (30.9%) | 0.293 |
| Smoker | 104 (14.3%) | 62 (12.9%) | 42 (17.2%) | 0.114* | 51 (15.7%) | 53 (13.2%) | 0.344 | 95 (14.7%) | 9 (11.1%) | 0.381 |
| Ex-Smoker | 300 (41.3%) | 205 (42.5%) | 95 (38.9%) | 0.353 | 124 (38.2%) | 176 (43.9%) | 0.119* | 271 (42.0%) | 29 (35.8%) | 0.284 |
| Mobility impairment^ | 238 (32.9%) | 175 (36.5%) | 63 (25.8%) | 0.004** | 97 (29.8%) | 141 (35.4%) | 0.112* | 204 (31.8%) | 34 (42.0%) | 0.066* |
| Vision impairment^ | 110 (15.2%) | 79 (16.4%) | 31 (12.8%) | 0.0194* | 43 (44.8%) | 400 (55.2%) | 0.195* | 98 (15.2%) | 12 (14.8%) | 0.920 |
| **Past foot treatment** | 726 |  |  |  |  |  |  |  |  |  |
| Yes | 252 (34.7%) | 180 (37.3%) | 72 (29.5%) | 0.036* | 107 (32.9%) | 145 (36.2%) | 0.362 | 217 (33.6%) | 35 (43.2%) | 0.088* |
| Podiatry | 178 (24.5%) | 137 (28.4%) | 41 (16.8%) | 0.001** | 67 (20.6%) | 111 (27.7%) | 0.028** | 152 (23.6%) | 26 (32.1%) | 0.092* |
| GP | 91 (12.5%) | 59 (12.2%) | 32 (13.1%) | 0.737 | 42 (12.9%) | 49 (12.2%) | 0.776 | 81 (12.6%) | 10 (12.3%) | 0.957 |
| Surgeon | 35 (4.8%) | 20 (4.1%) | 15 (6.1%) | 0.235 | 22 (6.8%) | 13 (3.2%) | 0.027** | 28 (4.3%) | 7 (8.6%) | 0.089* |
| Specialist Physician | 21 (2.9%) | 13 (2.7%) | 8 (3.3%) | 0.659 | 14 (4.3%) | 7 (1.3%) | 0.041** | 15 (2.3%) | 6 (7.4%) | 0.010** |
| Nurse | 19 (2.6%) | 12 (2.5%) | 7 (2.9%) | 0.762 | 11 (3.4%) | 8 (2.0%) | 0.244 | 15 (2.3%) | 4 (4.9%) | 0.165* |
| Orthotist | 4 (0.6%) | 2 (0.4%) | 2 (0.8%) | 0.495 | 2 (0.6%) | 2 (0.5%) | 0.833 | 4 (0.6%) | 0 | 1.000 |
| Other | 9 (1.2%) | 5 (1.0%) | 4 (1.6%) | 0.489 | 5 (1.5%) | 4 (1.0%) | 0.512 | 8 (1.2%) | 1 (1.2%) | 1.000 |
| **Foot-related conditions** | 726 |  |  |  |  |  |  |  |  |  |
| Amputation history | 34 (4.7%) | 24 (5.0%) | 10 (4.1%) | 0.596 | 16 (4.9%) | 18 (4.5%) | 0.783 | 28 (4.3%) | 6 (7.4%) | 0.218 |
| Foot ulcer history^ | 87 (12.0%) | 62 (12.9%) | 25 (10.2%) | 0.301 | 38 (11.7%) | 49 (12.3%) | 0.818 | 74 (11.5%) | 13 (16.0%) | 0.234 |
| Peripheral neuropathy^ | 159 (22.0%) | 118 (24.6%) | 41 (16.8%) | 0.017** | 68 (20.9%) | 91 (22.8%) | 0.543 | 132 (20.5%) | 27 (33.3%) | 0.009** |
| Foot deformity^ | 157 (22.4%) | 118 (25.1%) | 39 (16.8%) | 0.013** | 61 (19.5%) | 96 (24.7%) | 0.101* | 135 (21.7%) | 22 (27.2%) | 0.271 |
| PAD severity |  |  |  | 0.250 |  |  | 0.451 |  |  | 0.467 |
| Nil PAD | 572 (79.0%) | 369 (76.9%) | 203 (83.2%) |  | 265 (81.5%) | 307 (76.9%) |  | 510 (79.2%) | 62 (76.5%) |  |
| Mild PAD | 69 (9.5%) | 51 (10.6%) | 18 (7.4%) |  | 29 (8.9%) | 40 (10.0%) |  | 58 (9.0%) | 11 (13.6%) |  |
| Moderate PAD | 50 (6.9%) | 37 (7.7%) | 13 (5.3%) |  | 19 (5.8%) | 31 (7.8%) |  | 44 (6.8%) | 6 (7.4%) |  |
| Critical PAD | 33 (4.6%) | 23 (4.8%) | 10 (4.1%) |  | 12 (3.7%) | 21 (5.3%) |  | 31 (4.8%) | 2 (2.5%) |  |

**p* < 0.2; ***p* < 0.05; ^Variable has minor missing data (n<3); ^^n=702; GP: General Practitioner; PAD: Peripheral Arterial Disease; SD: standard deviation
