# Supplementary material for: Factors associated with type of footwear worn inside the house: a cross-sectional study
Source: J Foot Ankle Res. 2019 Aug 23;12:45. doi: 10.1186/s13047-019-0356-8 (PMC6708142; doi:10.1186/s13047-019-0356-8)
Supplement: Supplementary file 2 — Table S2. Characteristics and univariate analysis for those participants mostly wearing the indoor footwear types of barefoot, slippers, thongs/flip flops. (DOCX 78 kb) [file 13047_2019_356_MOESM2_ESM.docx]

**Additional file 2: Table S2:** Characteristics and univariate analysis for those participants mostly wearing the indoor footwear types of barefoot, slippers, thongs/flip flops

| Variables | All | Barefoot | | | Slippers | | | Thongs/ Flip Flops | | |
| --- | --- | --- | --- | --- | --- | --- | --- | --- | --- | --- |
|  |  | No | Yes | *p* Value | No | Yes | *p* Value | No | Yes | *p* Value |
| **Participants** | 726 | 507 (70.2%) | 219 (30.2%) |  | 571 (78.7%) | 155 (21.3%) |  | 621 (85.5%) | 105 (14.5%) |  |
| **Socio-demographics** | 725 |  |  |  |  |  |  |  |  |  |
| Age (SD) or [IQR] years | 65(18) | 68 [56-79] | 57 [41-69] | <0.001** | 62 [46-74] | 78 [66-84] | <0.001** | 63 (18) | 53 (16) | <0.001** |
| Male sex^ | 403 (55.7%) | 275 (54.3%) | 128 (58.7%) | 0.278 | 310 (54.5%) | 93 (60.0%) | 0.220 | 338 (54.5%) | 65 (62.5%) | 0.129* |
| Indigenous | 34 (4.7%) | 25 (4.9%) | 9 (4.1%) | 0.627 | 29 (5.1%) | 5 (3.2%) | 0.331 | 24 (3.9%) | 10 (9.6%) | 0.020** |
| Born overseas^ | 161 (22.2%) | 115 (22.8%) | 46 (21.0%) | 0.599 | 134 (23.6%) | 27 (17.4%) | 0.104* | 136 (21.9%) | 25 (24.3%) | 0.592 |
| <Year 10 Education Level^ | 390 (53.9%) | 285 (56.3%) | 105 (48.2%) | 0.043** | 281 (49.4%) | 109 (70.3%) | <0.001** | 330 (53.2%) | 60 (57.7%) | 0.398 |
| Socioeconomic Status | 705 |  |  | 0.009** |  |  | <0.001** |  |  | 0.270 |
| Most disadvantaged | 101 (14.3%) | 64 (13.0%) | 37 (17.5%) |  | 86 (15.5%) | 15 (10.0%) |  | 83 (13.8%) | 18 (17.6%) |  |
| Second most disadvantaged | 157 (22.3%) | 101 (20.4%) | 56 (26.5%) |  | 116 (20.9%) | 41 (27.3%) |  | 139 (23.1%) | 18 (17.6%) |  |
| Middle | 97 (13.8%) | 73 (14.8%) | 24 (11.4%) |  | 81 (14.6%) | 16 (10.7%) |  | 79 (13.1%) | 18 (17.6%) |  |
| Second least disadvantaged | 238 (33.8%) | 184 (37.2%) | 54 (25.6%) |  | 170 (30.6%) | 68 (45.3%) |  | 208 (34.5%) | 30 (29.4%) |  |
| Least disadvantaged | 112 (15.9%) | 72 (14.6%) | 40 (19.0% |  | 102 (18.4%) | 10 (6.7%) |  | 94 (15.6%) | 18 (17.6%) |  |
| Geographic Remoteness | 705 |  |  | 0.372 |  |  | 0.297 |  |  | 0.002** |
| Major city | 430 (61.0%) | 313 (63.4%) | 117 (55.5%) |  | 335 (60.4%) | 95 (63.3%) |  | 378 (62.7%) | 52 (51.0%) |  |
| Inner regional area | 152 (21.6%) | 100 (20.2%) | 52 (24.6%) |  | 120 (21.6%) | 32 (21.3%) |  | 132 (21.9%) | 20 (19.6%) |  |
| Outer regional area | 66 (9.4%) | 43 (8.7%) | 23 (10.9%) |  | 49 (8.8%) | 17 (11.3%) |  | 54 (9.0%) | 12 (11.8%) |  |
| Remote area | 30 (4.3%) | 19 (3.8%) | 11 (5.2%) |  | 27 (4.9%) | 3 (2.0%) |  | 20 (3.3%) | 10 (9.8%) |  |
| Very remote area | 27 (3.8%) | 19 (3.8%) | 8 (3.8%) |  | 24 (4.3%) | 3 (2.0%) |  | 19 (3.2%) | 8 (7.8%) |  |
| **Medical condition history** | 726 |  |  |  |  |  |  |  |  |  |
| Diabetes | 171 (23.6%) | 118 (23.3%) | 53 (24.2%) | 0.787 | 122 (21.4%) | 49 (31.6%) | 0.008** | 466 (25.0%) | 16 (15.2%) | 0.030** |
| Hypertension | 354 (48.8%) | 261 (51.5%) | 93 (42.5%) | 0.026** | 268 (46.9%) | 86 (55.5%) | 0.059* | 310 (49.9%) | 44 (41.9%) | 0.129* |
| Dyslipidaemia | 233 (32.1%) | 174 (34.3%) | 59 (26.9%) | 0.051* | 175 (30.6%) | 58 (37.4%) | 0.109* | 197 (31.7%) | 36 (34.3%) | 0.603 |
| Myocardial Infarct | 145 (20.5%) | 110 (21.7%) | 35 (16.0%) | 0.077* | 105 (18.4%) | 40 (25.8%) | 0.041** | 129 (20.8%) | 16 (15.2%) | 0.190* |
| Cerebrovascular Accident | 85 (11.7%) | 75 (14.8%) | 10 (4.6%) | <0.001** | 60 (10.5%) | 25 (16.1%) | 0.054* | 79 (12.7%) | 6 (5.7%) | 0.039** |
| Chronic Kidney Disease | 88 (12.1%) | 68 (13.4%) | 20 (9.1%) | 0.105* | 57 (10.0%) | 31 (20.5%) | 0.001** | 80 (12.9%) | 8 (7.6%) | 0.126* |
| Cancer | 171 (23.6%) | 122 (24.1%) | 49 (22.4%) | 0.623 | 127 (22.2%) | 44 (28.4%) | 0.110* | 148 (23.8%) | 23 (21.9%) | 0.667 |
| Arthritis | 270 (37.2%) | 203 (40.0%) | 67 (30.6%) | 0.016** | 195 (34.2%) | 75 (48.4%) | 0.001** | 237 (38.2%) | 33 (31.4%) | 0.187* |
| Depression | 189 (26.0%) | 131 (25.8%) | 58 (26.5%) | 0.856 | 150 (26.3%) | 39 (25.2%) | 0.780 | 161 (25.9%) | 28 (26.7%) | 0.873 |
| Smoker | 104 (14.3%) | 65 (12.8%) | 39 (17.8%) | 0.078* | 96 (16.8%) | 8 (5.2%) | <0.001** | 76 (12.2%) | 28 (26.7%) | <0.001** |
| Ex-Smoker | 300 (41.3%) | 213 (42.0%) | 87 (39.7%) | 0.566 | 228 (39.9%) | 72 (46.5%) | 0.144* | 254 (40.9%) | 46 (43.8%) | 0.576 |
| Mobility impairment^ | 238 (32.9%) | 184 (36.5%) | 54 (24.7%) | 0.002** | 164 (28.8%) | 74 (48.1%) | <0.001** | 226 (36.5%) | 12 (11.7%) | <0.001** |
| Vision impairment^ | 110 (15.2%) | 86 (17.0%) | 24 (11.0%) | 0.040** | 73 (12.8%) | 37 (23.9%) | 0.001** | 102 (16.5%) | 8 (7.7%) | 0.021** |
| **Past foot treatment** | 726 |  |  |  |  |  |  |  |  |  |
| Yes | 252 (34.7%) | 190 (37.5%) | 62 (28.3%) | 0.017** | 189 (33.1%) | 63 (40.6%) | 0.080* | 233 (37.5%) | 19 (18.1%) | <0.001** |
| Podiatry | 178 (24.5%) | 145 (28.6%) | 33 (15.1%) | <0.001** | 127 (22.2%) | 51 (32.9%) | 0.006** | 167 (26.9%) | 11 (10.5%) | <0.001** |
| GP | 91 (12.5%) | 64 (12.6%) | 27 (12.3%) | 0.912 | 70 (12.3%) | 21 (13.5%) | 0.667 | 82 (13.2%) | 9 (8.6%) | 0.185* |
| Surgeon | 35 (4.8%) | 22 (4.3%) | 13 (5.9%) | 0.357 | 28 (4.9%) | 7 (4.5%) | 0.842 | 35 (5.6%) | 0 | 0.006** |
| Specialist Physician | 21 (2.9%) | 14 (2.8%) | 7 (3.2%) | 0.748 | 17 (3.0%) | 4 (2.6%) | 1.000 | 20 (3.2%) | 1 (1.0%) | 0.342 |
| Nurse | 19 (2.6%) | 13 (2.6%) | 6 (2.7%) | 0.892 | 16 (2.8%) | 3 (1.8%) | 0.778 | 19 (3.1%) | 0 | 0.093* |
| Orthotist | 4 (0.6%) | 3 (0.6%) | 1 (0.5%) | 1.000 | 3 (0.5%) | 1 (0.6%) | 1.000 | 4 (0.6%) | 0 | 1.000 |
| Other | 9 (1.2%) | 6 (1.2%) | 3 (1.45) | 1.000 | 8 (1.4%) | 1 (0.6%) | 0.693 | 8 (1.3%) | 1 (1.0%) | 1.000 |
| **Foot-related conditions** | 726 |  |  |  |  |  |  |  |  |  |
| Amputation history | 34 (4.7%) | 27 (5.3%) | 7 (3.2%) | 0.213 | 24 (4.2%) | 10 (6.5%) | 0.240 | 39 (4.8%) | 4 (3.8%) | 0.806 |
| Foot ulcer history^ | 87 (12.0%) | 66 (13.0%) | 21 (9.6%) | 0.189* | 66 (11.6%) | 21 (13.5%) | 0.503 | 76 (12.3%) | 11 (10.5%) | 0.603 |
| Peripheral neuropathy^ | 159 (22.0%) | 121 (24.0%) | 38 (17.4%) | 0.048** | 119 (20.9%) | 40 (26.0%) | 0.175* | 144 (23.2%) | 15 (14.4%) | 0.045** |
| Foot deformity^ | 157 (22.4%) | 122 (24.7%) | 35 (16.7%) | 0.020** | 108 (19.6%) | 49 (32.5%) | 0.001** | 146 (24.3%) | 11 (10.9%) | 0.003** |
| PAD severity |  |  |  | 0.155* |  |  | 0.007** |  |  | 0.069* |
| Nil PAD | 572 (79.0%) | 388 (76.8%) | 184 (84.0%) |  | 464 (81.5%) | 108 (69.7%) |  | 481 (77.6%) | 91 (87.5%) |  |
| Mild PAD | 69 (9.5%) | 55 (10.9%) | 14 (6.4%) |  | 51 (9.0%) | 18 (11.6%) |  | 66 (10.6%) | 3 (2.9%) |  |
| Moderate PAD | 50 (6.9%) | 37 (7.3%) | 13 (3.7%) |  | 33 (5.8%) | 17 (11.0%) |  | 44 (7.1%) | 6 (5.8%) |  |
| Critical PAD | 33 (4.6%) | 25 (5.0%) | 8 (3.7%) |  | 21 (3.7%) | 12 (1.7%) |  | 29 (4.7%) | 4 (3.8%) |  |

**p* < 0.2; ***p* < 0.05; ^Variable has minor missing data (n<3); ^^n=702; GP: General Practitioner; PAD: Peripheral Arterial Disease; SD: standard deviation
