# Supplementary material for: Factors associated with type of footwear worn inside the house: a cross-sectional study
Source: J Foot Ankle Res. 2019 Aug 23;12:45. doi: 10.1186/s13047-019-0356-8 (PMC6708142; doi:10.1186/s13047-019-0356-8)
Supplement: Supplementary file 3 — Table S3. Characteristics and univariate analysis for those participants mostly wearing the indoor footwear types of backless slippers, moccasins, walking shoes. (DOCX 78 kb) [file 13047_2019_356_MOESM3_ESM.docx]

**Additional file 3: Table S3:** Characteristics and univariate analysis for those participants mostly wearing the indoor footwear types of backless slippers, moccasins, walking shoes

| Variables | All | Backless slippers | | | Moccasins | | | Walking shoes | | |
| --- | --- | --- | --- | --- | --- | --- | --- | --- | --- | --- |
|  |  | No | Yes | *p* Value | No | Yes | *p* Value | No | Yes | *p* Value |
| **Participants** | 726 | 672 (92.6%) | 54 (7.4%) |  | 692 (95.3%) | 34 (4.7%) |  | 695 (95.7%) | 31 (4.3%) |  |
| **Socio-demographics** | 725 |  |  |  |  |  |  |  |  |  |
| Age (SD) or [IQR] years | 65(18) | 64 [40-76] | 70 [58-76] | 0.129* | 62 (19) | 70 (10) | 0.009** | 65 [50-76] | 72 [59-81] | 0.134* |
| Male sex^ | 403 (55.7%) | 381 (56.9^) | 22 (40.7%) | 0.022** | 385 (55.8%) | 18 (52.9%) | 0.743 | 386 (55.7%) | 17 (54.8%) | 0.925 |
| Indigenous | 34 (4.7%) | 32 (4.8%) | 2 (3.7%) | 1.000 | 34 (4.9%) | 0 | 0.397 | 32 (4.6%) | 2 (6.5%) | 0.652 |
| Born overseas^ | 161 (22.2%) | 139 (20.7%) | 22 (40.7%) | 0.001** | 153 (22.2%) | 8 (23.5%) | 0.853 | 155 (22.4%) | 6 (19.4%) | 0.693 |
| <Year 10 Education Level^ | 390 (53.9%) | 365 (54.5%) | 25 (46.3%) | 0.246 | 372 (53.9%) | 18 (52.9%) | 0.912 | 377 (54.4%) | 13 (41.9%) | 0.173* |
| Socioeconomic Status | 705 |  |  | 0.921 |  |  | 0.604 |  |  | 0.049** |
| Most disadvantaged | 101 (14.3%) | 93 (14.3%) | 8 (15.1%) |  | 96 (14.3%) | 5 (15.6%) |  | 100 (14.8%) | 1 (3.3%) |  |
| Second most disadvantaged | 157 (22.3%) | 147 (22.5%) | 10 (18.9%) |  | 151 (22.4%) | 6 (18.8%) |  | 151 (22.4%) | 6 (20.0%) |  |
| Middle | 97 (13.8%) | 89 (13.7%) | 8 (15.1%) |  | 90 (13.4%) | 7 (21.9%) |  | 88 (13.0%) | 9 (30.0%) |  |
| Second least disadvantaged | 238 (33.8%) | 218 (33.4%) | 20 (37.7%) |  | 227 (33.7%) | 11 (34.4%) |  | 230 (34.1%) | 8 (26.7%) |  |
| Least disadvantaged | 112 (15.9%) | 105 (16.1%) | 7 (13.2%) |  | 109 (16.2%) | 3 (9.4%) |  | 106 (15.7%) | 6 (20.0%) |  |
| Geographic Remoteness | 705 |  |  | 0.991 |  |  | 0.997 |  |  | 0.744 |
| Major city | 430 (61.0%) | 397 (60.9%) | 33 (62.3%) |  | 410 (60.9%) | 20 (62.5%) |  | 409 (60.6%) | 21 (70.0%) |  |
| Inner regional area | 152 (21.6%) | 140 (21.5%) | 12 (22.6%) |  | 145 (21.5%) | 7 (21.9%) |  | 146 (21.6%) | 6 (20.0%) |  |
| Outer regional area | 66 (9.4%) | 62 (9.5%) | 4 (7.5%) |  | 63 (9.4%) | 3 (9.4%) |  | 64 (9.5%) | 2 (6.7%) |  |
| Remote area | 30 (4.3%) | 28 (4.3%) | 2 (3.8%) |  | 29 (4.3%) | 1 (3.1%) |  | 29 (4.3%) | 1 (3.3%) |  |
| Very remote area | 27 (3.8%) | 25 (3.8%) | 2 (3.8%) |  | 26 (3.9%) | 1 (3.1%) |  | 27 (4.0%) | 0 |  |
| **Medical condition history** | 726 |  |  |  |  |  |  |  |  |  |
| Diabetes | 171 (23.6%) | 160 (23.8%) | 11 (20.5%) | 0.567 | 164 (23.7%) | 7 (20.6%) | 0.676 | 162 (23.3%) | 9 (29.0%) | 0.463 |
| Hypertension | 354 (48.8%) | 318 (47.3%) | 36 (66.7%) | 0.006** | 336 (48.6%) | 18 (52.9%) | 0.617 | 338 (48.6%) | 16 (51.6%) | 0.745 |
| Dyslipidaemia | 233 (32.1%) | 218 (32.4%) | 15 (27.8%) | 0.480 | 216 (31.2%) | 17 (50.0%) | 0.022** | 221 (31.8%) | 12 (38.7%) | 0.420 |
| Myocardial Infarct | 145 (20.5%) | 136 (20.2%) | 9 (16.7%) | 0.528 | 136 (19.7%) | 9 (26.5%) | 0.332 | 136 (19.6%) | 9 (29.0%) | 0.197* |
| Cerebrovascular Accident | 85 (11.7%) | 76 (11.3%) | 9 (16.7%) | 0.239 | 77 (11.1%) | 8 (23.5%) | 0.049** | 80 (11.5%) | 5 (16.1%) | 0.395 |
| Chronic Kidney Disease | 88 (12.1%) | 81 (12.1%) | 7 (13.0%) | 0.844 | 86 (12.4%) | 2 (5.9%) | 0.416 | 82 (11.8%) | 6 (19.4%) | 0.207 |
| Cancer | 171 (23.6%) | 157 (23.4%) | 14 (25.9%) | 0.669 | 162 (23.4%) | 9 (26.5%) | 0.681 | 164 (23.6%) | 7 (22.6%) | 0.896 |
| Arthritis | 270 (37.2%) | 251 (37.4%) | 19 (35.2%) | 0.751 | 254 (36.7%) | 16 (47.1%) | 0.223 | 256 (36.8%) | 14 (45.2%) | 0.348 |
| Depression | 189 (26.0%) | 178 (26.5%) | 11 (20.4%) | 0.324 | 182 (26.3%) | 7 (20.6%) | 0.459 | 175 (25.2%) | 14 (45.2%) | 0.013** |
| Smoker | 104 (14.3%) | 99 (14.7%) | 5 (9.3%) | 0.269 | 101 (14.6%) | 3 (8.8%) | 0.457 | 100 (14.4%) | 4 (12.9%) | 1.000 |
| Ex-Smoker | 300 (41.3%) | 283 (42.1%) | 17 (31.5%) | 0.127* | 282 (40.8%) | 18 (52.9%) | 0.159* | 291 (41.9%) | 9 (29.0%) | 0.156* |
| Mobility impairment^ | 238 (32.9%) | 222 (33.2%) | 16 (29.6%) | 0.593 | 224 (32.5%) | 14 (41.2%) | 0.294 | 220 (31.8%) | 18 (58.1%) | 0.002** |
| Vision impairment^ | 110 (15.2%) | 103 (15.4%) | 7 (13.0%) | 0.635 | 103 (14.9%) | 7 (20.6%) | 0.369 | 108 (15.6%) | 2 (6.5%) | 0.207 |
| **Past foot treatment** | 726 |  |  |  |  |  |  |  |  |  |
| Yes | 252 (34.7%) | 226 (33.6%) | 26 (48.1%) | 0.031** | 239 (34.5%) | 13 (38.2%) | 0.658 | 236 (34.0%) | 16 (51.6%) | 0.043** |
| Podiatry | 178 (24.5%) | 159 (23.7%) | 19 (35.2%) | 0.058* | 168 (24.3%0 | 10 (29.4%) | 0.497 | 167 (24.0%) | 11 (35.5%) | 0.147* |
| GP | 91 (12.5%) | 84 (12.5%) | 7 (13.0%) | 0.921 | 86 (12.4%) | 5 (14.7%) | 0.603 | 85 (12.2%) | 6 (19.4%) | 0.262 |
| Surgeon | 35 (4.8%) | 32 (4.8%) | 3 (5.6%) | 0.739 | 34 (4.9%) | 1 (2.9%) | 1.000 | 33 (4.7%) | 2 (6.5%) | 0.657 |
| Specialist Physician | 21 (2.9%) | 21 (3.1%) | 0 | 0.393 | 19 (2.7%) | 2 (.59%) | 0.258 | 17 (2.4%) | 4 (12.9%) | 0.010** |
| Nurse | 19 (2.6%) | 17 (2.5%) | 2 (3.7%) | 0.646 | 18 (2.6%) | 1 (2.9%) | 0.603 | 16 (2.3%) | 3 (9.7%) | 0.043** |
| Orthotist | 4 (0.6%) | 4 (0.6%) | 0 | 1.000 | 3 (0.4%) | 1 (2.9%) | 0.175* | 4 (0.6%) | 0 | 1.000 |
| Other | 9 (1.2%) | 8 (1.2%) | 1 (1.9%) | 0.503 | 9 (1.3%) | 0 | 1.000 | 8 (1.2%) | 1 (3.2%) | 0.326 |
| **Foot-related conditions** | 726 |  |  |  |  |  |  |  |  |  |
| Amputation history | 34 (4.7%) | 32 (4.8%) | 2 (3.7%) | 1.000 | 33 (4.8%) | 1 (2.9%) | 1.000 | 32 (4.6%) | 2 (6.5%) | 0.652 |
| Foot ulcer history^ | 87 (12.0%) | 81 (12.1%) | 6 (11.1%) | 0.834 | 84 (12.2%) | 3 (8.8%) | 0.787 | 81 (11.7%) | 6 (19.4%) | 0.250 |
| Peripheral neuropathy^ | 159 (22.0%) | 147 (21.9%) | 12 (22.2%) | 0.962 | 151 (21.9%) | 8 (23.5%) | 0.821 | 148 (21.4%) | 11 (35.%) | 0.063* |
| Foot deformity^ | 157 (22.4%) | 142 (21.8%) | 15 (28.8%) | 0.244 | 153 (22.9%) | 4 (11.8%) | 0.128* | 148 (22.1%) | 9 (29.0%) | 0.362 |
| PAD severity |  |  |  | 0.443 |  |  | 0.081* |  |  | 0.078* |
| Nil PAD | 572 (79.0%) | 530 (79.1%) | 42 (77.8%) |  | 550 (79.7%) | 22 (64.7%) |  | 550 (79.4%) | 22 (71.0%) |  |
| Mild PAD | 69 (9.5%) | 61 (9.1%) | 8 (14.8%) |  | 63 (9.1%) | 6 (17.6%) |  | 62 (8.9%) | 7 (22.6%) |  |
| Moderate PAD | 50 (6.9%) | 48 (7.2%) | 2 (3.7%) |  | 45 (6.5%) | 5 (14.7%) |  | 49 (7.1%) | 1 (3.2%) |  |
| Critical PAD | 33 (4.6%) | 31 (4.6%) | 2 (3.7%) |  | 32 (4.6%) | 1 (2.9%) |  | 32 (4.6%) | 1 (3.2%) |  |

**p* < 0.2; ***p* < 0.05; ^Variable has minor missing data (n<3); ^^n=702; GP: General Practitioner; PAD: Peripheral Arterial Disease; SD: standard deviation
