# Supplementary material for: Factors associated with type of footwear worn inside the house: a cross-sectional study
Source: J Foot Ankle Res. 2019 Aug 23;12:45. doi: 10.1186/s13047-019-0356-8 (PMC6708142; doi:10.1186/s13047-019-0356-8)
Supplement: Supplementary file 4 — Table S4. Characteristics and univariate analysis for those participants mostly wearing the indoor footwear types of sandals, running shoes, socks only. (DOCX 77 kb) [file 13047_2019_356_MOESM4_ESM.docx]

**Additional file 4: Table S4:** Characteristics and univariate analysis for those participants mostly wearing the indoor footwear types of sandals, running shoes, socks only

| Variables | All | Sandals | | | Running shoes | | | Socks only | | |
| --- | --- | --- | --- | --- | --- | --- | --- | --- | --- | --- |
|  |  | No | Yes | *p* Value | No | Yes | *p* Value | No | Yes | *p* Value |
| **Participants** | 726 | 696 (95.9%) | 30 (4.1%) |  | 698 (96.1%) | 28 (3.9%) |  | 701 (96.4%) | 25 (3.4%) |  |
| **Socio-demographics** | 725 |  |  |  |  |  |  |  |  |  |
| Age (SD) or [IQR] years | 65(18) | 65 [50-76] | 73 [57-81] | 0.042** | 62 (19) | 59 (19) | 0.364 | 62 (19) | 64 (19) | 0.527 |
| Male sex^ | 403 (55.7%) | 393 (56.6%) | 10 (33.3%) | 0.012** | 383 (55.0%) | 20 (71.4%) | 0.087* | 393 (56.2%) | 10 (40.0%) | 0.109* |
| Indigenous | 34 (4.7%) | 34 (4.9%) | 0 | 0.391 | 33 (4.7%) | 1 (3.6%) | 1.000 | 31 (4.4%) | 3 (12.0%) | 0.107* |
| Born overseas^ | 161 (22.2%) | 157 (22.6%) | 4 (13.3%) | 0.231 | 155 (22.3%) | 6 (21.4%) | 0.916 | 156 (22.3%) | 5 (20.0%) | 0.784 |
| <Year 10 Education Level^ | 390 (53.9%) | 375 (54.0%) | 15 (50.0%) | 0.664 | 378 (54.3%) | 12 (42.9%) | 0.233 | 378 (54.1%) | 12 (48.0%) | 0.549 |
| Socioeconomic Status | 705 |  |  | 0.855 |  |  | 0.590 |  |  | 0.513 |
| Most disadvantaged | 101 (14.3%) | 96 (14.2%) | 5 (16.7%) |  | 98 (14.5%) | 3 (11.1%) |  | 97 (14.3%) | 4 (16.0%) |  |
| Second most disadvantaged | 157 (22.3%) | 151 (22.4%) | 6 (20.0%) |  | 151 (22.3%) | 6 (22.2%) |  | 154 (22.6%) | 3 (12.0%) |  |
| Middle | 97 (13.8%) | 91 (13.5%) | 6 (20.0%) |  | 95 (14.0%) | 2 (7.4%) |  | 95 (14.0%) | 2 (8.0%) |  |
| Second least disadvantaged | 238 (33.8%) | 229 (33.9%) | 9 (30.0%) |  | 229 (33.8%) | 9 (33.3%) |  | 228 (33.5%) | 10 (40.0%) |  |
| Least disadvantaged | 112 (15.9%) | 108 (16.0%) | 4 (13.3%) |  | 105 (15.5%) | 7 (25.9%) |  | 106 (15.6%) | 6 (24.0%) |  |
| Geographic Remoteness | 705 |  |  | 0.834 |  |  | 0.042** |  |  | 0.119* |
| Major city | 430 (61.0%) | 413 (61.2%) | 17 (56.7%) |  | 406 (59.9%) | 24 (88.9%) |  | 410 (60.3%) | 20 (80.0%) |  |
| Inner regional area | 152 (21.6%) | 143 (21.2%) | 9 (30.0%) |  | 149 (22.0%) | 3 (11.1%) |  | 149 (21.9%) | 3 (12.0%) |  |
| Outer regional area | 66 (9.4%) | 64 (9.5%) | 2 (6.7%) |  | 66 (9.7%) | 0 |  | 66 (9.7%) | 0 |  |
| Remote area | 30 (4.3%) | 29 (4.3%) | 1 (3.3%) |  | 30 (4.4%) | 0 |  | 30 (4.4%) | 0 |  |
| Very remote area | 27 (3.8%) | 26 (3.9%) | 1 (3.3%) |  | 27 (4.0%) | 0 |  | 25 (3.7%) | 2 (8.0%) |  |
| **Medical condition history** | 726 |  |  |  |  |  |  |  |  |  |
| Diabetes | 171 (23.6%) | 166 (23.9%) | 5 (16.7%) | 0.364 | 168 (24.1%) | 3 (10.7%) | 0.102* | 168 (24.0%) | 3 (12.0%) | 0.166* |
| Hypertension | 354 (48.8%) | 337 (48.4%) | 17 (56.7%) | 0.376 | 245 (49.4%) | 9 (32.1%) | 0.073* | 340 (48.5%) | 14 (56.0%) | 0.461 |
| Dyslipidaemia | 233 (32.1%) | 224 (32.2%) | 9 (30.0%) | 0.802 | 225 (32.2%) | 8 (28.6%) | 0.684 | 228 (32.5%) | 5 (20.0%) | 0.187* |
| Myocardial Infarct | 145 (20.5%) | 136 (19.5%) | 9 (30.0%) | 0.161* | 143 (20.5%) | 2 (7.1%) | 0.083* | 141 (20.1%) | 4 (16.0%) | 0.801 |
| Cerebrovascular Accident | 85 (11.7%) | 80 (11.5%) | 5 (16.7%) | 0.382 | 77 (11.0%) | 8 (28.6%) | 0.011** | 80 (11.4%) | 5 (20.0%) | 0.200 |
| Chronic Kidney Disease | 88 (12.1%) | 85 (12.2%) | 3 (10.0%) | 1.000 | 87 (12.5%) | 1 (3.6%) | 0.236 | 88 (12.6%) | 0 | 0.061* |
| Cancer | 171 (23.6%) | 164 (23.6%) | 7 (23.3%) | 0.977 | 164 (23.5%) | 7 (25.0%) | 0.854 | 165 (23.5%) | 6 (24.0%) | 0.957 |
| Arthritis | 270 (37.2%) | 256 (36.8%) | 14 (46.7%) | 0.273 | 265 (38.0%) | 5 (17.9%) | 0.031* | 261 (37.2%) | 9 (36.0%) | 0.900 |
| Depression | 189 (26.0%) | 176 (25.3%) | 13 (43.3%) | 0.027** | 183 (26.2%) | 6 (21.4%) | 0.571 | 188 (26.8%) | 1 (4.0%) | 0.011** |
| Smoker | 104 (14.3%) | 100 (14.4%) | 4 (13.3%) | 1.000 | 101 (14.5%) | 3 (10.7%) | 0.785 | 101 (14.4%) | 3 (12.0%) | 1.000 |
| Ex-Smoker | 300 (41.3%) | 286 (41.1%) | 14 (46.7%) | 0.544 | 287 (41.1%) | 13 (46.4%) | 0.576 | 292 (41.7%) | 8 (32.0%) | 0.335 |
| Mobility impairment^ | 238 (32.9%) | 224 (32.3%) | 14 (46.7%) | 0.102* | 226 (32.5%) | 12 (42.9%) | 0.254 | 229 (32.8%) | 9 (36.0%) | 0.739 |
| Vision impairment^ | 110 (15.2%) | 106 (15.3%) | 4 (13.3%) | 1.000 | 104 (14.9%) | 6 (21.4%) | 0.416 | 103 (14.7%) | 7 (28.0%) | 0.085* |
| **Past foot treatment** | 726 |  |  |  |  |  |  |  |  |  |
| Yes | 252 (34.7%) | 238 (34.2%) | 14 (46.7%) | 0.160* | 241 (34.5%) | 11 (39.3%) | 0.604 | 242 (34.5%) | 10 (40.0%) | 0.572 |
| Podiatry | 178 (24.5%) | 167 (24.0%) | 11 (36.7%) | 0.114* | 171 (24.5%) | 7 (35.0%) | 0.952 | 170 (24.3%) | 8 (32.0%) | 0.376 |
| GP | 91 (12.5%) | 87 (12.5%) | 4 (13.3%) | 0.782 | 88 (12.6%) | 3 (10.7%) | 1.000 | 86 (12.3%) | 5 (20.0%) | 0.227 |
| Surgeon | 35 (4.8%) | 34 (4.9%) | 1 (3.3%) | 1.000 | 32 (4.6%) | 3 (10.7%) | 0.147* | 33 (4.7%) | 2 (8.0%) | 0.342 |
| Specialist Physician | 21 (2.9%) | 21 (3.0%) | 0 | 1.000 | 21 (3.0%) | 0 | 1.000 | 20 (2.9%) | 1 (.40%) | 0.526 |
| Nurse | 19 (2.6%) | 19 (2.7%) | 0 | 1.000 | 19 (2.7%) | 0 | 1.000 | 18 (2.6%) | 1 (4.0%) | 0.491 |
| Orthotist | 4 (0.6%) | 4 (0.6%) | 0 | 1.000 | 4 (0.6%) | 0 | 1.000 | 3 (0.4%) | 1 (04.0%) | 0.131* |
| Other | 9 (1.2%) | 8 (1.1%) | 1 (3.3%) | 0.317 | 9 (1.3%) | 0 | 1.000 | 8 (1.1%) | 4.0%) | 0.272 |
| **Foot-related conditions** | 726 |  |  |  |  |  |  |  |  |  |
| Amputation history | 34 (4.7%) | 34 (4.9%) | 0 | 0.391 | 33 (4.7%) | 1 (3.6%) | 1.000 | 31 (4.4%) | 3 (12.0%) | 0.107* |
| Foot ulcer history^ | 87 (12.0%) | 84 (12.1%) | 3 (10.3%) | 1.000 | 84 (12.1%) | 3 (10.7%) | 1.000 | 83 (11.9%) | 4 (16.0%) | 0.527 |
| Peripheral neuropathy^ | 159 (22.0%) | 150 (21.6%) | 9 (30.0%) | 0.277 | 149 (21.4%) | 10 (35.7%) | 0.073* | 156 (22.3%) | 3 (12.0%) | 0.221 |
| Foot deformity^ | 157 (22.4%) | 147 (21.8%) | 10 (34.5%) | 0.110* | 148 (22.0%) | 9 (32.1%) | 0.205 | 153 (22.5%) | 4 (17.4%) | 0.561 |
| PAD severity |  |  |  | 0.878 |  |  | 0.459 |  |  | 0.305 |
| Nil PAD | 572 (79.0%) | 548 (78.8%) | 24 (82.8%) |  | 551 (79.2%) | 21 (75.0%) |  | 553 (79.1%) | 19 (76.0%) |  |
| Mild PAD | 69 (9.5%) | 66 (9.5%) | 3 (10.3%) |  | 67 (9.6%) | 2 (7.1%) |  | 65 (9.3%) | 4 (16.0%) |  |
| Moderate PAD | 50 (6.9%) | 49 (7.1%) | 1 (3.4%) |  | 46 (6.6%) | 4 (14.3%) |  | 50 (7.2%) | 0 |  |
| Critical PAD | 33 (4.6%) | 32 (4.6%) | 1 (.34%) |  | 32 (4.6%) | 1 (3.6%) |  | 31 (4.4%) | 2 (8.0%) |  |

**p* < 0.2; ***p* < 0.05; ^Variable has minor missing data (n<3); ^^n=702; GP: General Practitioner; PAD: Peripheral Arterial Disease; SD: standard deviation
