# Supplementary material for: Factors associated with type of footwear worn inside the house: a cross-sectional study
Source: J Foot Ankle Res. 2019 Aug 23;12:45. doi: 10.1186/s13047-019-0356-8 (PMC6708142; doi:10.1186/s13047-019-0356-8)
Supplement: Supplementary file 5 — Table S5. Characteristics and univariate analysis for those participants mostly wearing the indoor footwear types of ugg boots, oxford shoes. (DOCX 25 kb) [file 13047_2019_356_MOESM5_ESM.docx]

**Additional file 5: Table S5:** Characteristics and univariate analysis for those participants mostly wearing the indoor footwear types of ugg boots, oxford shoes

| Variables | All | Ugg Boots | | | Oxford shoes | | |
| --- | --- | --- | --- | --- | --- | --- | --- |
|  |  | No | Yes | *p* Value | No | Yes | *p* Value |
| **Participants** | 726 | 707 (97.4%) | 19 (2.6%) |  | 715 (98.5%) | 11 (1.5%) |  |
| **Socio-demographics** | 725 |  |  |  |  |  |  |
| Age (SD) or [IQR] years | 65(18) | 62 (19) | 56 (20) | 0.188* | 62 (19) | 67 (16) | 0.359 |
| Male sex^ | 403 (55.7%) | 397 (56.3%) | 6 (31.6%) | 0.032** | 396 (55.5%) | 7 (63.6%) | 0.763 |
| Indigenous | 34 (4.7%) | 33 (4.7%) | 1 (5.3%) | 0.603 | 33 (4.6%) | 1 (9.1%) | 0.413 |
| Born overseas^ | 161 (22.2%) | 159 (22.6%) | 2 (10.5%) | 0.273 | 155 (21.7%) | 6 (54.5%) | 0.019** |
| <Year 10 Education Level^ | 390 (53.9%) | 381 (54.0%) | 9 (47.4%) | 0.565 | 385 (54.0%) | 5 (45.5%) | 0.573 |
| Socioeconomic Status | 705 |  |  | 0.493 |  |  | 0.375 |
| Most disadvantaged | 101 (14.3%) | 97 (14.1%) | 4 (21.1%) |  | 100 (14.4%) | 1 (9.1%) |  |
| Second most disadvantaged | 157 (22.3%) | 155 (22.6%) | 2 (10.5%) |  | 156 (22.5%) | 1 (9.1%) |  |
| Middle | 97 (13.8%) | 95 (13.8%) | 2 (10.5%) |  | 96 (13.8%) | 1 (9.1%) |  |
| Second least disadvantaged | 238 (33.8%) | 229 (33.4%) | 9 (47.4%) |  | 234 (33.7%) | 4 (36.4%) |  |
| Least disadvantaged | 112 (15.9%) | 110 (16.0%) | 2 (10.5%) |  | 108 (15.6%) | 4 (36.4%) |  |
| Geographic Remoteness | 705 |  |  | 0.694 |  |  | 0.644 |
| Major city | 430 (61.0%) | 419 (61.1%) | 11 (57.9%) |  | 421 (60.7%) | 9 (81.8%) |  |
| Inner regional area | 152 (21.6%) | 146 (21.3%) | 6 (31.6%) |  | 151 (21.8%) | 1 (9.1%) |  |
| Outer regional area | 66 (9.4%) | 65 (9.5%) | 1 (5.3%) |  | 65 (9.4%) | 1 (9.1%) |  |
| Remote area | 30 (4.3%) | 30 (4.4%) | 0 |  | 30 (4.3%) | 0 |  |
| Very remote area | 27 (3.8%) | 26 (3.8%) | 1 (5.3%) |  | 27 (3.9%) | 0 |  |
| **Medical condition history** | 726 |  |  |  |  |  |  |
| Diabetes | 171 (23.6%) | 164 (23.2%) | 7 (36.8%) | 0.175* | 168 (23.5%) | 3 (27.3%) | 0.727 |
| Hypertension | 354 (48.8%) | 347 (49.1%) | 7 (36.8%) | 0.292 | 350 (49.0%) | 4 (36.4%) | 0.407 |
| Dyslipidaemia | 233 (32.1%) | 227 (32.1%) | 6 (31.6%) | 0.961 | 231 (32.3%) | 2 (18.2%) | 0.517 |
| Myocardial Infarct | 145 (20.5%) | 140 (19.8%) | 5 (26.3%) | 0.559 | 142 (19.9%) | 3 (27.3%) | 0.466 |
| Cerebrovascular Accident | 85 (11.7%) | 84 (11.9%) | 1 (5.3%) | 0.714 | 84 (11.7%) | 1 (9.1%) | 1.000 |
| Chronic Kidney Disease | 88 (12.1%) | 83 (11.7%) | 5 (26.3%) | 0.068* | 86 (12.0%) | 2 (18.2%) | 0.632 |
| Cancer | 171 (23.6%) | 169 (23.9%) | 2 (10.5%) | 0.272 | 170 (23.8%) | 1 (9.1%) | 0.473 |
| Arthritis | 270 (37.2%) | 260 (36.8%) | 10 (52.6%) | 0.158* | 268 (37.5%) | 2 (18.2%) | 0.226 |
| Depression | 189 (26.0%) | 182 (25.7%) | 7 (36.8%) | 0.292 | 187 (26.2%) | 2 (18.2%) | 0.737 |
| Smoker | 104 (14.3%) | 99 (14.0%) | 5 (26.3%) | 0.173* | 104 (14.5%) | 0 | 0.380 |
| Ex-Smoker | 300 (41.3%) | 293 (41.4%) | 7 (36.8%) | 0.688 | 295 (41.3%) | 5 (45.5%) | 0.768 |
| Mobility impairment^ | 238 (32.9%) | 231 (32.8%) | 7 (36.8%) | 0.712 | 236 (33.1%) | 2 (18.2%) | 0..519 |
| Vision impairment^ | 110 (15.2%) | 106 (15.0%) | 4 (21.1%) | 0.512 | 108 (15.1%) | 2 918.2%) | 0.678 |
| **Past foot treatment** | 726 |  |  |  |  |  |  |
| Yes | 252 (34.7%) | 243 (34.4%) | 9 (47.4%) | 0.240 | 249 (34.8%) | 3 (27.3%) | 0.756 |
| Podiatry | 178 (24.5%) | 170 (24.0%) | 8 (42.1%) | 0.100 | 175 (24.5%) | 3 (27.3%) | 0.736 |
| GP | 91 (12.5%) | 88 (12.4%) | 3 (15.8%) | 0.721 | 91 (12.7%) | 0 | 0.376 |
| Surgeon | 35 (4.8%) | 34 (4.8%) | 1 (5.3%) | 0.614 | 35 (4.9%) | 0 | 1.000 |
| Specialist Physician | 21 (2.9%) | 21 (3.0%) | 0 | 1.000 | 21 (2.9%) | 0 | 1.000 |
| Nurse | 19 (2.6%) | 17 (2.4%) | 2 (10.5%) | 0.085 | 19 (2.7%) | 0 | 1.000 |
| Orthotist | 4 (0.6%) | 4 (0.6%) | 0 | 1.000 | 4 (0.6%) | 0 | 1.000 |
| Other | 9 (1.2%) | 9 (1.3%) | 0 | 1.000 | 9 (1.3%) | 0 | 1.000 |
| **Foot-related conditions** | 726 |  |  |  |  |  |  |
| Amputation history | 34 (4.7%) | 33 (4.7%) | 1 (5.3%) | 0.603 | 33 (4.6%) | 1 (9.1%) | 0.412 |
| Foot ulcer history^ | 87 (12.0%) | 82 (11.6%) | 5 (26.3%) | 0.066* | 86 (12.0%) | 1 (9.1%) | 1.000 |
| Peripheral neuropathy^ | 159 (22.0%) | 153 (21.7%) | 6 (31.6%) | 0.397 | 158 (22.2%) | 1 (9.1%) | 0.471 |
| Foot deformity^ | 157 (22.4%) | 152 (22.2%) | 5 (27.8%) | 0.570 | 156 (22.6%) | 1 (9.1%) | 0.471 |
| PAD severity |  |  |  | 0.570 |  |  | 0.686 |
| Nil PAD | 572 (79.0%) | 555 (78.7%) | 17 (89.5%) |  | 562 (78.8%) | 10 (0.9%) |  |
| Mild PAD | 69 (9.5%) | 68 (9.6%) | 1 (5.3%) |  | 68 (9.5%) | 1 (9.1%) |  |
| Moderate PAD | 50 (6.9%) | 50 (7.1%) | 0 |  | 50 (7.0%) | 0 |  |
| Critical PAD | 33 (4.6%) | 32 (4.5%) | 1 (5.3%) |  | 33 (4.6%) | 0 |  |

**p* < 0.2; ***p* < 0.05; ^Variable has minor missing data (n<3); ^^n=702; GP: General Practitioner; PAD: Peripheral Arterial Disease; SD: standard deviation
